# Interplay of IL‐17A/IL‐17RA signaling with microbial homeostasis in systemic anti‐tumoral responses

**DOI:** 10.1002/mco2.524

**Published:** 2024-04-07

**Authors:** Shi‐jun He, Jian‐ping Zuo, Ze‐min Lin

**Affiliations:** ^1^ Laboratory of Immunopharmacology, State Key Laboratory of Drug Research, Shanghai Institute of Materia Medica, Chinese Academy of Sciences Shanghai China; ^2^ Innovation Research Institute of Traditional Chinese Medicine, Shanghai University of Traditional Chinese Medicine Shanghai China; ^3^ University of Chinese Academy of Sciences Beijing China

## Abstract

Enteric IL‐17RA deficiency leads to gut dysbiosis, consequently initiating the proliferation of tumors at remote locations. The deficiency or blockade of enteric IL‐17RA induces the secretion of IL‐17A by B cells and Th17 cells in response to microbial signals, resulting in a systemic elevation of IL‐17A and fostering the growth of remote tumors. This figure was created with BioRender.com.

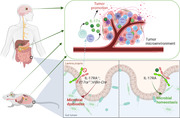

1

The recent study by Chandra and colleagues, published in *Cancer Cell*, sheds light on the significant impact of microbes, specifically through interleukin‐17A (IL‐17A) signaling, on distant tumor behavior.^1^ This discovery implies that targeted microbial ablation holds promise as an effective strategy to surmount the constraints of pharmacological inhibition, addressing the intricate dynamics of the signaling of IL‐17A and its receptor (IL‐17RA) across various compartments in the context of the cancer therapy.

IL‐17A, a pivotal pro‐inflammatory cytokine primarily derived from T‐helper 17 (Th17) cells, was initially recognized for its essential role for its fundamental contribution to the host's defense mechanism against various microbes, notably extracellular fungi and bacteria.^2^, ^3^, ^4^ IL‐17RA is widely expressed across cell types, facilitating the potential reactivity of numerous cells to this cytokine, particularly emphasizing the analysis of non‐immune cell types, such as mesenchymal cells and epithelial located within tissues experiencing inflammation or disease. In terms of functionality, IL‐17A plays a contributory role in the pathogenesis of autoimmune disorders, encompassing conditions such as systemic lupus erythematosus, multiple sclerosis, rheumatoid arthritis, inflammatory bowel disease, and psoriasis.^1^, ^2^, ^5^ Apart from its involvement in autoimmune disorders, IL‐17A emerges as a key player in cancer, with notable implications in pancreatic ductal adenocarcinoma (PDAC), where IL‐17A/IL‐17RA signaling profoundly impacts various facets of tumor biology including cell proliferation, growth, progression, microenvironmental remodeling, and resistance to treatment, all mediated by the inflammatory milieu within the tumor microenvironment.^1^, ^4^, ^5^ Studies reveal context‐dependent effects of IL‐17A in cancer, demonstrating instances of Th17‐mediated anti‐tumor immunity and IL‐17A‐producing cells contributing to CD8^+^ T cell exhaustion in melanoma, ultimately enhancing the efficacy of immunotherapeutic approaches. Concurrently, the impact of the microbiota on PDAC progression has been observed in murine models and metastatic PDAC patients.^1^, ^3^ However, since 2015, the Food and Drug Administration (FDA) has authorized the use of IL‐17A‐targeting monoclonal antibodies for autoimmune diseases including psoriatic arthritis, plaque psoriasis, and ankylosing spondylitis. Nevertheless, efforts to impede IL‐17A signaling in inflammatory bowel disease, such as Crohn's disease, through the use of antibodies (such as secukinumab, brodalumab, or ixekizumab) have encountered constrained success, frequently resulting in the exacerbation of inflammation. Given the pivotal role of IL‐17A in bolstering the host's defense against extracellular bacterial and fungal infections, its microbial regulation may be a significant factor contributing to the clinical trial failures of IL‐17A‐targeting monoclonal antibodies.^1^, ^3^ Therefore, investigating enteric IL‐17A/IL‐17RA signaling and its regulation by microorganisms in the context of distant tumors becomes a vital avenue of research to better address the challenges posed by IL‐17‐related therapeutic interventions.

Chandra and colleagues embarked on a study to explore the impact of extra‐tumoral IL‐17A/IL‐17RA signaling on the growth of pancreatic tumors, leading to the observation of unexpected outcomes associated with IL‐17RA deficiency. Contrary to expectations, global IL‐17RA deficiency (*Il17ra^−/−^*) markedly accelerated tumor progression in both subcutaneous and orthotopic PDAC models. Transcriptomic profiling revealed a distinct pattern, characterized by the upregulation of genes related to IL‐17A signaling in tumors, while these genes were downregulated in the ilea of *Il17ra^−/−^* mice. Strikingly, IL‐17A and IL‐17F levels surged in the circulation of *Il17ra^−/−^* mice, coinciding with an observed state of microbial dysbiosis. In investigating the correlation between microbial dysbiosis and tumor growth, the researchers synergistically combined ampicillin with anti‐IL‐17RA antibodies, resulting in an observed enhanced antitumor effect. Notably, ampicillin alone emulated the effects of a broad‐spectrum antibiotic cocktail in reducing systemic IL‐17A levels. The pivotal finding of the study elucidated that extra‐tumoral IL‐17RA deficiency or blockade unexpectedly elicited the secretion of IL‐17A in response to microbial stimuli, subsequently leading to the systemic elevation of IL‐17A levels and promoting the growth of pancreatic tumors.

To further elucidate the consequences of enteric IL‐17RA deficiency and its systemic impact, Chandra and colleagues progressed beyond the unexpected findings associated with global IL‐17RA deficiency. Their investigation delved into the systemic ramifications of disrupted IL‐17A/IL‐17RA signaling in the intestine, targeting both pancreatic and syngeneic murine glioma (GBM) tumors. *Il17rafl/fl; Villin‐Cre mice*, featuring targeted enteric deletion of IL‐17RA, exhibited the most pronounced tumor growth, coupled with discernible neurological manifestations and symptoms.

Building on the unexpected outcomes of global IL‐17RA deficiency, Chandra and colleagues then explored the systemic impact of disrupted IL‐17A/IL‐17RA signaling in the intestine, focusing on both pancreatic and syngeneic murine glioma (GBM) tumors. *Il17ra^fl/fl^*; *Villin‐Cre* mice, with specific enteric deletion of IL‐17RA, exhibited the most aggressive tumor growth, accompanied by notable neurological signs and symptoms (Figure 1). The investigation unveiled a notable augmentation in B cells, which emerged as a crucial immune response facilitating tumor progression. By employing single‐cell sequencing (scRNA‐seq) analysis, the study elucidated the cellular origins of both IL‐17A and IL‐17F within tumors, identifying Th17 cells and B cells as key contributors. This delineated a complex interplay between the gut microbiota, IL‐17A signaling, and the tumor microenvironment. In addition, dual oxidase 2 (DUOX2), a gene whose expression is differentially modulated by IL‐17A, was primarily detected in tumor cell clusters derived from mice with IL‐17RA deficiency. The examination of pathways highlighted the involvement of DUOX2 in critical cellular functions such as Wnt signaling, DNA damage response, catalytic activity, and hydrolase activity. Subsequent functional experiments provided confirmation of IL‐17A‐mediated regulation of DUOX2 in pancreatic cancer cells.^1^

**FIGURE 1:**
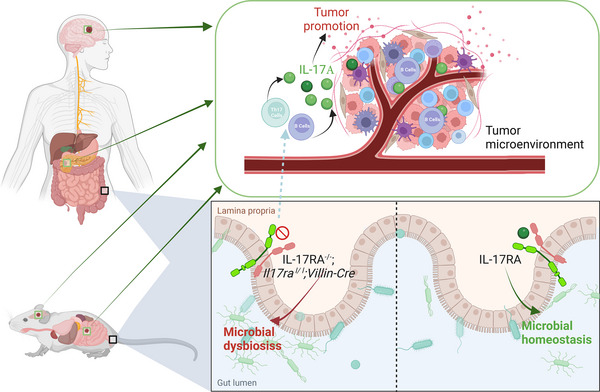
Enteric IL‐17RA deficiency leads to gut dysbiosis, consequently initiating the proliferation of tumors at remote locations. The deficiency or blockade of enteric IL‐17RA induces the secretion of IL‐17A by B cells and Th17 cells in response to microbial signals, resulting in a systemic elevation of IL‐17A and fostering the growth of remote tumors. This figure was created with BioRender.com. IL, Interleukin.

In elucidating clinical implications and future research directions, Chandra and colleagues extended its implications to human PDAC, demonstrating the association of IL‐17A expression with T cells and elevated IL‐17A levels in PDAC patients.^1^ Significantly, increased DUOX2 expression in PDAC was positively associated with IL‐17A expression, implying potential clinical relevance. Pathway analysis revealed diminished responses to oxygen‐containing compounds, lipopolysaccharide, and bacteria in PDAC with elevated DUOX2 expression, highlighting the potential involvement of IL‐17A signaling in modulating interactions between cancer and the microbiota.

In their discussion, Chandra and colleagues scrutinized the clinical implications of IL‐17A inhibition, emphasizing the presence of an antagonistic IL‐17A‐mediated reaction among patients and the plausible pro‐tumorigenic impacts associated with the prolonged IL‐17A inhibitor therapy.^1^ The cellular of IL‐17A, particularly T cells and B cells, were identified as playing key roles in tumor growth, with a specific focus on the involvement of IL‐17F. The study emphasizes the significance of DUOX2 in pancreatic cancer, establishing a connection between its expression, microbial defense, and IL‐17A levels. The authors put forward the concept of future clinical trials that combine IL‐17A inhibition with microbial interventions to enhance effectiveness and mitigate the influence of tumor‐promoting microbes (Figure 1).

In conclusion, Chandra and colleagues present a comprehensive investigation into the intricate relationship between microbiota, IL‐17A signaling, and tumor growth.^1^ The unexpected outcomes of global and enteric IL‐17RA deficiency highlight the delicate balance within the mIL‐17A/IL‐17RA signaling axis. The study underscores the clinical relevance of IL‐17A inhibition, shedding light on potential resistance mechanisms and the need for a nuanced approach in the cancer therapy. By unraveling the intricate impact of IL‐17A signaling on microbial homeostasis, this study lays the groundwork for future research and therapeutic approaches aimed at fully leveraging IL‐17A‐targeting interventions in the cancer treatment.

## AUTHOR CONTRIBUTIONS

S.J.H. and J.P.Z. conceived and drafted the initial draft of the manuscript, Z.M.L drew the figure, and approved the final version of the article. All authors have read and approved the final manuscript.

## CONFLICT OF INTEREST STATEMENT

The authors declare no competing interests.

## ETHICS STATEMENT

Not applicable.

## Data Availability

Not applicable.
